# The Effects of Combined Scutellaria and Saffron Supplementation on Mood Regulation in Participants with Mild-to-Moderate Depressive Symptoms: A Randomized, Double-Blind, Placebo-Controlled Study

**DOI:** 10.3390/nu17050809

**Published:** 2025-02-26

**Authors:** Valérie Dormal, Marlène Suchareau, Sylvie Copine, Laurent Simar, Louise Deldicque

**Affiliations:** 1 Center of Investigation in Clinical Nutrition, Université Catholique de Louvain, Rue du Marathon, 3, B-1348 Louvain-la-Neuve, Belgium; sylvie.copine@uclouvain.be (S.C.); docteur.simar@outlook.com (L.S.); louise.deldicque@uclouvain.be (L.D.); 2 Institute of Neuroscience, Université Catholique de Louvain, B-1348 Louvain-la-Neuve, Belgium; 3 Comercial Quimica Masso, Viladomat, 321, 08029 Barcelona, Spain; msuchareau@cqmasso.com

**Keywords:** saffron, scutellaria, depression, anxiety, mood regulation, BDI, clinical trial

## Abstract

**Background/Objectives:** The global prevalence of mental health disorders, particularly anxiety and depression, has increased significantly, with rates further elevated by the COVID-19 pandemic. Conventional pharmacological treatments, while effective, often lead to side effects that can impact patient adherence and quality of life. This has driven interest in safer, natural adjunctive therapies. *Crocus sativus* L. (*Iridaceae*) (saffron) and *Scutellaria baicalensis Georgi* (*Lamiaceae*) (scutellaria) have individually shown potential, in humans and animals, respectively, as mood regulators, with bioactive compounds that modulate neurotransmitter systems and possess anti-inflammatory and anxiolytic effects. This study aimed (1) to explore the efficacy and safety of scutellaria extracts in humans and (2) to test a possible synergistic effect when combining scutellaria and saffron on mood regulation in individuals experiencing mild-to-moderate depressive symptoms. **Methods:** In a randomized, double-blind, placebo-controlled trial, 180 participants with mild-to-moderate depressive symptoms were assigned to receive either scutellaria extract alone (SCUTELL’UP^®^), saffron extract alone (SAFFR’ACTIV^®^), a combination of scutellaria and saffron extracts (SAFFR’UP^®^), or a placebo for six weeks. The primary outcome was assessed using a standardized depression scale (Beck Depression Inventory). The secondary outcomes, including anxiety, emotional state, well-being level, and sleep quality, were all assessed using validated questionnaires. Safety and tolerability were evaluated throughout the study period. **Results:** The results confirmed the beneficial effects of saffron extract on depressive and anxious symptoms, as well as its role in improving sleep quality. For the first time in humans, scutellaria extract demonstrated a positive effect on mood regulation. Furthermore, a synergistic effect of the combination of these two extracts was identified, leading to enhanced improvements in depressive and anxious symptoms and emotional well-being among individuals with mild-to-moderate depression, compared to the placebo group. Minimal adverse effects were reported across all treatment groups. **Conclusions:** This natural adjunctive nutritional strategy offers a promising alternative for individuals seeking safer options for mental health support. Further research is warranted to exclude potential long-term side effects and to explore potential mechanisms of this combined supplementation.

## 1. Introduction

In recent years, mental health disorders have become an increasingly prominent global health concern. The COVID-19 pandemic contributed to a substantial rise in stress, anxiety, and other mental health issues, as evidenced by numerous studies testifying to the sharp increase in stress-related disorders worldwide [1,2]. Simultaneously, a notable increase in demand has been observed for natural products designed to improve sleep quality and promote relaxation [3,4]. Despite the availability of conventional treatments for mental disorders, such as antidepressants, anxiolytics, and antipsychotics, these options are often associated with various side effects that can impact patients’ quality of life and adherence to treatment [5,6]. Additionally, the use of conventional antidepressant drugs, like selective serotonin reuptake inhibitors (SSRIs) or tricyclic antidepressants (TCAs), is often characterized by incomplete therapeutic response, limited remission rates, high risk of relapse, poor tolerability, and frequent early discontinuation, highlighting the need for alternative or adjunctive therapeutic options with fewer negative consequences [5,7].

One of the most widely used herbal remedies for mood regulation is *Hypericum perforatum*, commonly known as St. John’s Wort [8]. While its antidepressant properties are well documented, its use, however, is limited by significant side effects, including photosensitivity and interactions with various medications, such as antidepressants, oral contraceptives, and cardiac drugs, due to its influence on liver enzymes [8,9]. One promising plant-based alternative is *Crocus sativus* L. (*Iridaceae*), commonly known as saffron, which has gained attention in recent years for its potential antidepressant and anxiolytic effects. Saffron contains bioactive compounds such as crocins, crocetin, and safranal, which have been shown to have mood-enhancing properties by inhibiting the reuptake of the neurotransmitters like norepinephrine, dopamine, and serotonin [7,10,11,12]. This inhibition helps sustain neurotransmitter levels in synaptic spaces, thereby increasing their overall concentration in the brain. Additionally, saffron has been found to elevate glutamate levels [13]. This mechanism of action mirrors that of commonly prescribed antidepressants [7]. Clinical studies have demonstrated that saffron supplementation can significantly reduce symptoms of depression and anxiety, with results comparable to those of conventional antidepressants in some cases (for a meta-analysis, see [14]). Notably, a six-week ingestion of 60 mg/day of saffron showed antidepressant effects equivalent to 100 mg/day of sertraline [15]. Beyond its mood-regulating properties, saffron has been found to improve sleep quality and reduce stress-related symptoms, offering a multi-faceted approach to mental health management (for a review and a meta-analysis, see [16,17], respectively). For instance, supplementation with 15.5 mg/day of saffron for six weeks improved sleep quality, sleep latency, and total sleep duration compared to placebo [18]. Additionally, an eight-week trial with 30 mg/day of saffron significantly reduced mild-to-moderate comorbid depression and anxiety in individuals with type 2 diabetes [19]. In a pilot study on children with ADHD, a three-month intervention with 30 mg/day of saffron effectively alleviated hyperactivity symptoms and improved sleep patterns [20]. Furthermore, saffron is generally well tolerated, with minimal side effects, making it a promising natural alternative for individuals seeking safer, adjunctive treatments for mood disorders.

Another plant with emerging evidence for mood regulation is *Scutellaria baicalensis Georgi* (*Lamiaceae*). Traditionally used in Chinese medicine, this herb contains flavonoids, particularly baicalin and baicalein, which have demonstrated anti-inflammatory, antioxidant, and anxiolytic effects [21,22]. Some pre-clinical studies in animal models suggest that *Scutellaria baicalensis* may help alleviate symptoms of anxiety and mild depression by acting as positive modulators of GABA_A receptors in a subtype-selective manner and reducing central nervous system inflammation (for reviews, see [23,24]). Additionally, *Scutellaria baicalensis* has shown potential in improving cognitive function and reducing stress in animals, which may further contribute to its therapeutic effects on mood disorders [25]. Importantly, *Scutellaria baicalensis* is generally well tolerated with a favorable safety profile in human [26], making it a promising candidate for natural interventions aimed at improving mental health, particularly in individuals who may experience adverse effects from conventional treatments. Further studies in humans are needed to demonstrate the true impact of *Scutellaria baicalensis* on anxiety and depression, as its effects have yet to be confirmed in human clinical trials.

Individually, the effects of *Scutellaria baicalensis* have been extensively studied in preclinical in vitro and in vivo models [23], while *Crocus sativus* has been evaluated in numerous clinical trials [7]; however, to the best of our knowledge, the effects and safety of their combination have never been investigated. Therefore, the aim of the present study was twofold: first, to confirm the efficacy and safety of saffron and scutellaria extracts individually in regulating mood in individuals with mild-to-moderate depressive symptoms and, second, to evaluate whether a synergistic effect exists when these two extracts are combined, through a randomized, double-blind, controlled trial.

## 2. Materials and Methods

### 2.1. Participants

**Figure 1:**
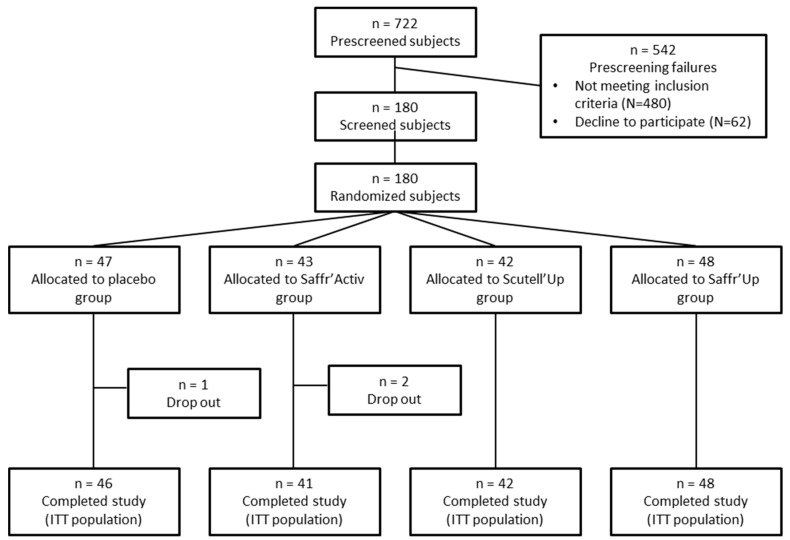
Flow chart of the study samples. The chart presents the number of participants who were screened, underwent randomization, completed the study treatment, and were analyzed for the primary and secondary outcomes. ITT, intention-to-treat.

A total of 722 participants were assessed for eligibility, and 180 participants were randomized between May 2022 and July 2024 into the Placebo (*n* = 47), the SCUTELL’UP (*n* = 42), the SAFFR’ACTIV (*n* = 43), or the SAFFR’UP (*n* = 48) groups (Figure 1). Three participants dropped out of the study (2 for personal reasons and 1 for medical reasons; 1 from the Placebo group and 2 from the SAFFR’ACTIV group). Participants were recruited by posters, mail, and social networks. To be included, the participants had to meet the following criteria: (1) being a woman or man aged between 18 and 75 years; (2) presenting a depressive episode, according to the DSM-5 definition; (3) experiencing mild-to-moderate depressive symptoms (scores ranging from 14 to 28 in the Beck Depression Inventory; BDI); (4) having had a recent depressive episode (less than 2 years); (5) speaking French; and (6) having stated willingness to comply with all study procedures and be available for the duration of the study. The participants were excluded if they presented one of the following exclusion criteria: (1) presenting a depressive disorder of another nature or any other mental pathology; (2) being at risk of suicide or having made a suicide attempt in the last 5 years; (3) having depression for more than 2 years; (4) undergoing psychotropic or psychotherapeutic treatment (current or in the month prior to inclusion); (5) having a serious health problem for which the investigator concluded that it was not in the participant’s interest to participate in the study; (6) using products containing piperine or millpertuis or those having a known effect on mood within the last 4 weeks; (7) being a woman of childbearing age who was pregnant or breastfeeding or who wished to become pregnant within the next 8 weeks; and (8) having an allergy or contraindication to any component of the study drug.

All selected participants provided written informed consent. This study was approved by the local ethical committee (Comité d’Ethique Hospitalo-Facultaire UCLouvain/Cliniques Universitaires Saint-Luc). The trial was carried out in accordance with the Declaration of Helsinki and the Good Clinical Practice as required by the following regulations: the Belgian law of 7 May 2004 regarding experiments on human beings and the EU Directive 2001/20/EC on Clinical Trials (registration at clinicaltrials.gov as NCT06138470).

### 2.2. Study Design

**Figure 2:**
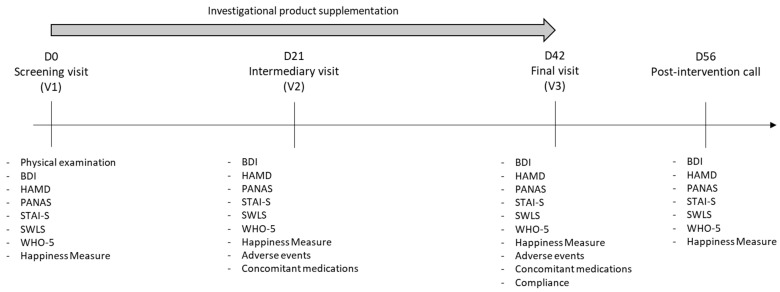
Schematic representation of the study design.

This interventional study is a double-blind, randomized, placebo-controlled parallel study. Pre-screening was proposed with an online questionnaire sent by email. Then, the screening visit (V1), comprising a physical examination (including measurement of body weight, height, and heart rate at rest) and an evaluation of depressive symptoms (using the BDI and Hamilton Depression Scale; HAMD) was organized (Figure 2). The participants who met all the criteria were randomly assigned to the Placebo, the SCUTELL’UP, the SAFFR’ACTIV, or the SAFFR’UP groups and invited to take the first capsule. Then, evaluation of psychological and emotional processing was carried out. Participants completed self-questionnaires assessing positive and negative affects (PANAS), anxiety level (State-Trait Anxiety Inventory; STAI-S), quality of life (Satisfaction with Life Scale; SWLS), and well-being level (Wellbeing index; WHO-5; and Happiness Measure). During the intervention, both groups were instructed to ingest one capsule with a glass of water each morning for the next 42 days. The capsule (of similar appearance) contained either 20 mg of *Scutellaria baicalensis* (SCUTELL’UP^®^ standardized at 90.04% baicalin (HPLC) that corresponds to 18.08 mg of baicalin) in the SCUTELL’UP group, 30 mg of *Crocus sativus* (SAFFR’ACTIV^®^ SAF 3C PIM standardized at 5.45% crocins (HPLC) and 2.6% safranal (UV, method ISO 3632-2 [27]) that corresponds to 1.635 mg of crocins analyzed by HPLC and 0.78 mg of safranal analyzed by UV, method ISO 3632-2) in the SAFFR’ACTIV group, 20 mg of *Scutellaria baicalensis* (SCUTELL’UP^®^ standardized at 90.04% baicalin (HPLC)) and 30 mg of *Crocus Sativus* (SAFFR’ACTIV^®^ standardized at 5.45% crocins (HPLC) and 2.6% safranal (UV, method ISO 3632-2)) in the SAFFR’UP group, and 450 mg of maltodextrin in the Placebo group. A second intermediary visit (V2) was scheduled 3 weeks (±7 days) after V1 (Figure 2). During this visit, concomitant medication and adverse events were recorded. Subjects were invited to complete the following questionnaires: BDI, PANAS, HAMD, STAI-S, SWLS, Happiness Measure, and WHO-5. A third visit (V3) was carried out 6 weeks (±7 days) after V1 (Figure 2). Concomitant medication and adverse events were also recorded. Questionnaires evaluating depression level and psychological and emotional processing were completed. Participants returned unused tablets for the evaluation of compliance. Finally, 2 weeks after the end of the intervention (Day 56 ± 7), subjects were invited to complete additional questionnaires (BDI, PANAS, HAMD, STAI-S, SWLS, Happiness Measure, WHO-5, presence of intercurrent visits and new treatments) to follow the evolution of symptoms and emotions (Figure 2).

### 2.3. Emotional and Psychological Processing Measures

The severity of depression was assessed by the BDI [28], which is a 21-question, multiple-choice, self-report inventory with high reliability in clinical and research contexts [24]. All items are scored on a 4-point scale, ranging from 0 to 3. HAM-D is a validated 17-item rating scale that has been widely applied in clinical trial studies to measure the severity of depressive symptoms [29]. Nine items are scored on a 5-point scale, ranging from 0 to 4, and eight are scored from 0 to 2. State anxiety level was measured by the STAI-S questionnaire, a psychological inventory based on a 4-point Likert scale and consisting of 20 questions on a self-report basis [30]. Emotional processing was evaluated by the PANAS [31], which is a self-report-scale-validated questionnaire [32] that consists of different words that describe feelings and emotions. In the PANAS questionnaire, 10 items evaluate the positive affect, whereas 10 items evaluate the negative affect. The French version of the SWLS [33], a self-administrated, 5-item, 7-point Likert scale was used to evaluate the cognitive component of well-being, while the first item from the Happiness Measure [34] assessed the affective component of well-being. Finally, the 5-item WHO-5 [35] was completed by the participants to measure subjective well-being. All these validated instruments were selected for their complementary focus on distinct dimensions of well-being [36,37], ensuring a comprehensive and standardized assessment relevant to mood regulation.

### 2.4. Statistical Analyses

Sample size was calculated based on the primary endpoint, i.e., differences in the adjusted BDI score between V1 and V3. Using the software PASS 14.0.7 and the multiple comparisons test, 45 participants per group (with an estimated 7% of dropout subjects) were needed to observe a decrease of 5 points in the BDI mean scores in any of the 3 intervention groups compared to the control. Standard deviation was set at 4, power at 80%, and alpha at 0.05.

Statistical analyses were performed using the software systems SPSS version 24 (IBM SPSS Statistics, Armonk, NY, USA). Baseline differences in demographic characteristics between the groups were examined using *χ*^2^ tests (for categorical variables) and One-Way ANOVAs (for continuous variables). Cronbach’s alpha values were calculated for STAI-S, PANAS, SWLS, Happiness Measure, and WHO-5 questionnaires at V1 to assess internal consistency. First, a repeated-measures ANOVA with Session (V1, V2 vs. V3) as the within-subject factor and groups (SAFFR’ACTIV, SCUTELL’UP, SAFFR’UP, vs. Placebo) as between-subject factors was conducted to explore time x treatment interactions. Then, the evolution of an endpoint between baseline and V2, baseline and V3, baseline and D56, V2 and V3, and V3 and D56, within a group, was evaluated using paired *t*-tests. These tests allow us to determine the intervention effect inside each group. The endpoint changes from baseline at each intervention period ([V2—baseline] or [V3—baseline] or [D56—baseline]) were compared between groups using a One-Way ANOVA. These tests allow us to determine the intervention effect between groups, considering the baseline. Moreover, to assess (1) the improvement between the intermediary visit and the final visit and (2) the evolution of symptoms just after the end of the intervention, the endpoint changes between (1) [V3–V2] and (2) [D56–V3] were also compared between groups using a One-Way ANOVA. Of note, for the D56 measurements, data from 19 participants are missing (8 from the placebo group, 2 from the SAFFR’ACTIV group, 6 from the SCUTELL’UP group, and 3 from the SAFFR’UP group) (Table S1). Additional exploratory analyses were carried out to evaluate the specific effects of the different plant extract interventions on sleep (HAMD sleep-related items) and the possible presence of a gender effect for the [V3–V2] BDI score difference (Tables S2 and S3). Data are shown as mean ± SD. To address multiple comparisons, the Bonferroni correction was applied by multiplying the obtained *p*-values by a factor of four, corresponding to the number of comparisons performed for each endpoint changes. Statistical significance was defined as *p* < 0.05 after correction.

## 3. Results

### 3.1. Baseline Characteristics

No differences in age, gender, body mass index (BMI), and heart rate at rest were found between the four groups before the start of the study (Table 1). There were also no differences in BDI and HAMD scores at baseline. Moreover, Cronbach’s alpha values calculated in our study sample for STAI-S, PANAS, WHO-5, and SWLS questionnaires at V1 were all superior to 0.8, indicating a good internal consistency and supporting the reliability of the measures in our sample.

### 3.2. Depression and Anxiety Assessment

**Figure 3:**
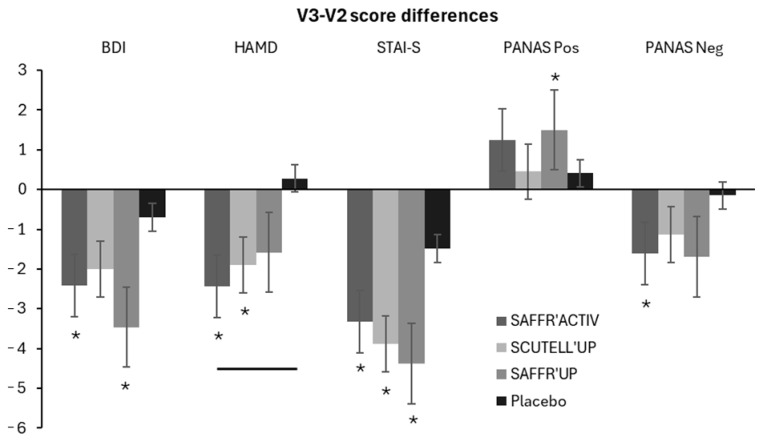
Depression, anxiety, and emotion assessment. Positive and negative score differences between V3 and V2 for the questionnaires BDI, HAMD, STAI-S, and PANAS in the SAFFR’ACTIV, SCUTTEL’UP, SAFFR’UP, and Placebo groups. Results are presented as the mean ± SD. An asterisk indicates a difference between V3 and V2 within a group (*p* < 0.05). A line indicates a difference between an intervention and the placebo group (*p* < 0.05).

The descriptive statistics for the questionnaire scores are presented in Table 2. For the BDI score, the ANOVA revealed the main effect of Session (*p* < 0.001, *η*^2^ = 0.454) but no significant Session × Group interaction (*p* > 0.05). After 21 (V2), 42 (V3), and 56 days (D56) of intervention, a decrease in the BDI score was observed compared to baseline (V1) in each group (*p* < 0.001). This decrease was not different between the four groups. Between V2 and V3, a decrease was observed only in the SAFFR’ACTIV (−2.4 ± 5.7; *p* = 0.036, Cohen’s *d* = 0.4) and the SAFFR’UP (−3.5 ± 6.6; *p* = 0.004, Cohen’s *d* = 0.5) groups (Figure 3). Post hoc *t*-tests comparing V3–V2 differences between each group revealed no difference between groups. However, when the V3–V2 difference was explored separately for men (*n* = 50) and women (*n* = 127), an improvement was observed specifically in women in the SAFFR’UP group (−3.6 ± 7.1; *p* = 0.020, Cohen’s *d* = 0.5; Table S3). Moreover, after 2 weeks of wash-out (D56), no differences were observed in either group between D56 and V3 (Table S1).

For the HAMD score, the ANOVA revealed the main effect of Session (*p* < 0.001, *η*^2^ = 0.594) and a Session × Group interaction (*p* = 0.050, *η*^2^ = 0.036). A reduction in HAMD scores was found in all groups at 21 (V2), 42 (V3), and 56 days (D56) of intervention compared to baseline (*p* < 0.001, Table 2 and Table S1). This decrease was not different between the four groups. Between V2 and V3, a reduction in HAMD scores was observed in the SAFFR’ACTIV (−2.4 ± 4.2; *p* = 0.004, Cohen’s *d* = 0.6) and SCUTELL’UP (−1.9 ± 4.9; *p* = 0.044, Cohen’s *d* = 0.4) groups, while no change was detected in the Placebo group (Figure 3). Post hoc *t*-tests comparing V3–V2 differences between each group revealed a larger decrease in the SAFFR’ACTIV (*p* = 0.024) group compared to the Placebo group. After 2 weeks of wash-out, no differences were observed in either group between D56 and V3 (Table S1). When focusing more specifically on the average of the three items of the HAMD questionnaire relating to sleep quality, a specific improvement was observed between V2 and V3 only for the SAFFR’ACTIV group (−0.15 ± 0.45; *p* = 0.033, Cohen’s *d* = 0.3; Table S2).

For the STAI-S score, the ANOVA revealed the main effect of Session (*p* < 0.001, *η*^2^ = 0.241) but no significant Session × Group interaction (*p* > 0.05). Compared to baseline, a decrease in the STAI-S score was observed after 21 days of intervention (V2) in all groups (*p* < 0.05), except for the SCUTELL’UP group (*p* = 0.50), and after both 42 (V3) and 56 (D56) days of intervention in all groups (*p* < 0.05) (Table 2 and Table S1). Between V2 and V3, a decrease in the STAI-S score was measured in the SAFFR’ACTIV (*p* = 0.008, Cohen’s *d* = 0.5), SCUTELL’UP (*p* = 0.048, Cohen’s *d* = 0.4), and SAFFR’UP (*p* = 0.012, Cohen’s *d* = 0.5) groups, but not in the Placebo group (Figure 3). Post hoc *t*-tests comparing the V3–V2 changes between groups revealed no difference between the four groups. Moreover, after 2 weeks of wash-out, no difference was observed in either group between D56 and V3 (Table S1).

### 3.3. Emotional Assessment

For the PANAS—Positive score, the ANOVA revealed the main effect of Session (*p* < 0.001, *η*^2^ = 0.135) but no significant Session × Group interaction (*p* > 0.05). Compared to baseline, an increase in the PANAS—Positive score was observed in each group (*p* < 0.05), except in the SCUTELL’UP group, after both 21 and 42 days of intervention (Table 2). At D56, the increase was maintained only in the SAFFR’ACTIV and the SCUTTEL’UP groups (*p* < 0.05) compared to baseline (Table S1). Between V2 and V3, an increase in the PANAS—Positive score was observed only in the SAFFR’UP (*p* = 0.042, Cohen’s *d* = 0.4) group (Figure 3). Post hoc *t*-tests comparing the V3–V2 changes between groups revealed no difference between the four groups. After 2 weeks of wash-out, no difference was observed in either group between D56 and V3, except for the Placebo group, in which the PANAS—Positive score decreased (*p* = 0.048, Cohen’s *d* = 0.4; Table S1).

For the PANAS—Negative score, the ANOVA revealed the main effect of Session (*p* < 0.001, *η*^2^ = 0.201) but no significant Session × Group interaction (*p* > 0.05). After both 21 days (V2) and 42 days (V3) of intervention, the PANAS—Negative score was reduced in all groups (*p* < 0.05) compared to baseline, with no difference between the four groups (Table 2). At D56, the decrease was maintained only in the SAFFRACT’IV and the placebo groups (*p* < 0.05) compared to baseline (Table S1). Between V2 and V3, a decrease was observed only in the SAFFR’ACTIV (*p* = 0.028, Cohen’s *d* = 0.4) group. Post hoc *t*-tests comparing the V3-V2 differences among the groups revealed no differences (Figure 3). After 2 weeks of wash-out, no difference was observed in either group between D56 and V3 (Table S1).

### 3.4. Well-Being Assessment

**Figure 4:**
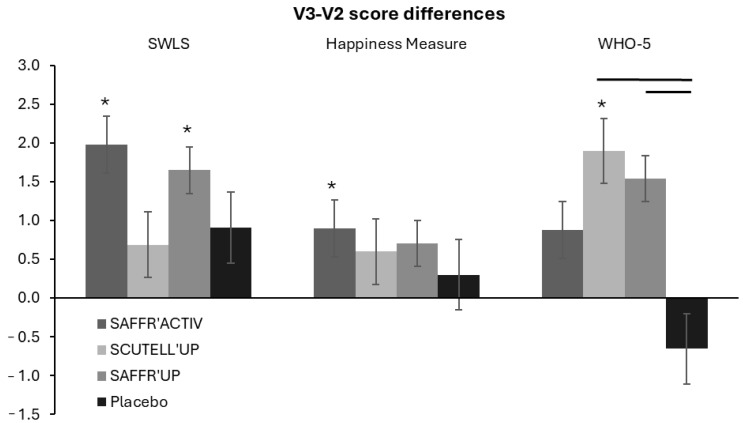
Well-being assessment. Score differences between V3 and V2 for the questionnaires SWSL, Happiness Measure, and WHO-5 in SAFFR’ACTIV, SCUTTEL’UP, SAFFR’UP, and Placebo groups. Results are presented as the mean ± SD. An asterisk indicates a difference between V3 and V2 within a group (*p* < 0.05). A line indicates a difference between an intervention and the placebo group (*p* < 0.05).

Regarding satisfaction with life, the ANOVA revealed the main effect of Session (*p* < 0.001, *η*^2^ = 0.227) and a significant Session × Group interaction (*p* = 0.043, *η*^2^ = 0.037). After 21 days of intervention (V2), only the Placebo and SAFFR’UP groups increased the SWLS score compared to baseline (*p* < 0.05), without any difference between those two groups (Table 2). After both 42 (V3) and 56 (D56) days of intervention, the SWLS score was increased in each group compared to baseline (*p* < 0.05, Table S1). Between V2 and V3, an increase was observed only in the SAFFR’ACTIV (*p* < 0.001, Cohen’s *d* = 0.6) and SAFFR’UP (*p* < 0.001, Cohen’s *d* = 0.6) groups (Figure 4). Post hoc *t*-tests comparing the V3-V2 differences among the groups revealed no difference between the four groups. After 2 weeks of wash-out, no difference was observed in either group between D56 and V3 (Table S1).

Concerning well-being, the ANOVA revealed the main effect of Session (*p* < 0.001, *η*^2^ = 0.369) but no significant Session × Group interaction (*p* > 0.05) for the Happiness score. After 21 (V2), 42 (V3), and 56 (D56) days of intervention, all groups increased their Happiness scores compared to baseline (*p* < 0.020), without any difference between the four groups (Table 2 and Table S1). Between V2 and V3, a decrease in the score was observed in the SAFFR’ACTIV (*p* < 0.001, Cohen’s *d* = 0.7) but not the Placebo groups (Figure 4). Post hoc *t*-tests comparing the V3-V2 differences among the groups revealed no difference between the four groups. After 2 weeks of wash-out, no difference was observed in either group between D56 and V3 (Table S1).

Finally, for the WHO-5 score, the ANOVA revealed the main effect of Session (*p* < 0.001, *η*^2^ = 0.331) but no significant Session × Group interaction (*p* > 0.05). After 21 (V2), 42 (V3), and 56 (D56) days of intervention, all groups increased their WHO-5 scores compared to baseline (*p* < 0.001), without any differences between the four groups (Table 2 and Table S1). Between V2 and V3, an increase was observed only in the SCUTELL’UP (*p* = 0.048, Cohen’s *d* = 0.5) group (Figure 4). Post hoc *t*-tests comparing the V3–V2 differences among the groups revealed that the increase in the SCUTELL’UP (*p* = 0.016) and SAFFR’UP (*p* = 0.04) groups was larger than in the Placebo group, which did not modify its score (Figure 4). After 2 weeks of wash-out, no difference was observed in either group between D56 and V3 (Table S1).

### 3.5. Safety and Compliance

The saffron and scutellaria extract supplementation was globally well tolerated. A total of 66 adverse events were reported during the study, with only 13 possibly related to the study product. Participants mainly experienced headaches (*n* = 11), respiratory infections (*n* = 21), sleeping disorders (*n* = 2), or gastrointestinal disorders (*n* = 7). Only three adverse events of severe intensity were reported (two in the Placebo group and one in the SCUTELL’UP group) and were not related to the study product (i.e., angina, COVID-19, and diverticulis). High compliance was observed in all groups with an intake of 99.1 ± 3.9% in the SAFFR’ACTIV group, 99.4 ± 4.4% in the SCUTELL’UP group, 99.4 ± 2.7% in the SAFFR’UP group, and 99.2 ± 4.6% in the Placebo group.

## 4. Discussion

The present study aimed to evaluate the effects of a standardized saffron extract (SAFFR’ACTIV^®^), a standardized scutellaria extract (SCUTELL’UP^®^), and the combination of those two extracts (SAFFR’UP^®^) on the regulation of mood in participants experiencing mild-to-moderate depressive symptoms. The main finding of the present study is the positive evolution of the score obtained via the questionnaires BDI, HAMD, PANAS—Negative, Happiness Measure, and WHO-5 in all groups, and in the STAI-S, PANAS—Positive, and SWLS questionnaires in almost all the groups, including the Placebo group, after 3 and 6 weeks of intervention. This placebo effect can probably be explained by the fact that the volunteers who took part in this clinical study were engaged in a process in which they were more willing to talk about their problems and to initiate a proactive desire to improve their well-being [38,39]. Even though a placebo effect was present, a further improvement of key variables was observed in the SAFFR’ACTIV, SCUTELL’UP, and SAFFR’UP groups compared to the Placebo group between the second and the third visits. These results indicate that this type of natural intervention based on plant extracts requires a certain period of time, i.e., at least 3 weeks, and regular intake before any beneficial effect appears.

More specifically, the key effects and variables favorably impacted by the saffron and scutellaria extracts may be summarized as follows: between V2 and V3 and compared to the Placebo, the decrease in the HAMD score was larger in the SAFFR’ACTIV group, and the increase in the WHO-5 score was larger in the SCUTELL’UP and SAFFR’UP groups. In addition, the following regulations were observed after supplementation but not after receiving the placebo, indicating a tendency towards a better effect of the supplementation over the placebo. This was the case for the decrease in PANAS—Negative, as well as the increase in the Happiness Measure between V2 and V3 in the SAFFR’ACTIV group, the decrease in STAI-S in all three intervention groups, the increase in PANAS—Positive in SAFFR’UP, the increase in HAMD in the SAFFR’ACTIV and SCUTELL’UP groups, and the increase in BDI and SWLS in both SAFFR’ACTIV and SAFFR’UP groups. Altogether, the combination of saffron and scutellaria extracts in the SAFFR’UP group seems to be an efficient supplementation, as it concomitantly reduced depression (according to the BDI questionnaire) and anxiety (according to the STAI-S questionnaire) symptoms, and it increased positive emotions (PANAS—Positive) to a further extent than the placebo. The effects of the saffron extract alone (SAFFR’ACTIV) and the scutellaria extract alone (SCUTELL’UP) are also significant, as the HAMD score was improved in both the SAFFR’ACTIV and SCUTELL’UP groups, and WHO-5 was improved in the SCUTELL’UP group, compared to the Placebo.

Our findings are in strong agreement with previous research demonstrating the positive effects of saffron on mood regulation and well-being at the dose and duration used here, i.e., 30 mg per day for 6 weeks. Ingestion of 30 mg of saffron extract per day for 8 weeks reduces the symptoms of postpartum depression [40], reduces the symptoms of depression among overweight women with mild-to-moderate depression after 12 weeks of supplementation [41], and has a similar effect as the selective serotonin reuptake inhibitor fluoxetine in the treatment of mild-to-moderate depression when taken for 6 weeks [42]. These studies, among others, validate the therapeutic potential of saffron at the studied dosage and make it a natural, evidence-based approach for mood regulation. Another important benefit of saffron, demonstrated in previous clinical trials, is its ability to improve anxiety. A daily dose of 30 mg of saffron extract, administered over 6 weeks, was found to be as effective as the selective serotonin reuptake inhibitor citalopram in treating major depressive disorder with anxious distress [43]. In teenagers and healthy adults, a daily dose of 28 mg of saffron extract, taken for 4 or 8 weeks, also reduced symptoms of anxiety and depression, improved mood, and enhanced stress management [44,45]. Using the STAI-S questionnaire in the present study, we confirm the effect of saffron extract on anxiety. Moreover, we also observed a specific effect of SAFFR’ACTIV^®^ on the three items related to sleep in the HAMD questionnaire. This result is in accordance with a previous clinical study having evidenced the beneficial effects of the same saffron extract on sleep quality [18]. The primary bioactive compounds in saffron include crocins, crocetin, picrocrocin, and safranal, with safranal and crocins identified as the main contributors to its mood-enhancing and anxiolytic effects [46]. Different mechanisms of action have been found, amongst which the ability of the aforementioned bioactive molecules to modulate key brain chemicals, including serotonin, dopamine, and glutamate, offer therapeutic benefits without the typical side effects associated with conventional antidepressants [10,12,13]. Additionally, different saffron extracts have been found to regulate the hypothalamic–pituitary–adrenal axis and GABA-A receptors, mechanisms linked to its anxiolytic properties [47,48,49,50], as well as reduce the secretion of the stress hormone corticosterone [49].

Looking at the anxiolytic and antidepressant effects of our scutellaria extract, we confirm for the first time in humans key findings from animal studies having investigated the effects of the bioactive flavonoids of scutellaria extract, including wogonin, baicalein, and baicalin [51,52,53,54,55,56,57,58]. Tests such as the sucrose preference test, the forced swimming test, and the tail suspension test consistently reported increased sucrose preference and/or decreased immobility time with baicalin compared to chronic unpredictable mild stress or chronic corticosterone groups [53,55,57,58]. Recent research has explored the mechanisms underlying the beneficial effects of scutellaria extract and its bioactive compounds on depression and anxiety. The primary target appears to be the dopamine system, which is implicated in various neurological and mental disorders [25]. Both in vitro and in vivo studies have shown that baicalin and baicalein, two major compounds in scutellaria extract, increase dopamine levels in the brain and protect dopaminergic neurons from mitochondrial and oxidative stress-related toxicity [25,59]. Baicalin has also been found to enhance synaptogenesis and regulate the GABAergic system, contributing to its anxiolytic effects [51]. Additionally, the bioactive compounds of scutellaria extract promote neurogenesis and neuronal differentiation, modulate hyperactivation of the hypothalamic–pituitary–adrenal axis, reduce lipid peroxidation, and counteract inflammation [59]. They also act as monoamine oxidase inhibitors, thereby enhancing the release of key neurotransmitters such as dopamine, norepinephrine, and serotonin, which play critical roles in mood regulation [21].

When combined together, our saffron and scutellaria extracts demonstrated a synergistic effect on the brain, particularly in reducing anxiety and depressive symptoms. This synergy is likely due to the complementary mechanisms of action of the two extracts. Furthermore, the combination was proven to be safe, with no serious adverse effects reported during the study. This demonstrates that the combined use of SAFFR’ACTIV^®^ and SCUTELL’UP^®^ not only amplifies therapeutic benefits but also maintains a favorable safety profile, making it a promising strategy for managing anxiety and depression.

The effect of a wash-out period was also evaluated in this study. After 2 weeks without supplementation (D56), no differences were observed compared to V3, except in the Placebo group, for the positive PANAS score. The maintenance of health benefits of saffron after a wash-out period has been explored in prior studies, particularly for ocular health. In a pilot study on patients with primary open-angle glaucoma, oral supplementation with 30 mg/day of aqueous saffron extract, as an adjunct to timolol and dorzolamide, induced significant effects three weeks after initiation, which returned to baseline one month after discontinuation [60]. Similarly, a crossover study on patients with early age-related macular degeneration observed a persistent effect of saffron even after transitioning to placebo for 6 months [61]. To our knowledge, this is the first study to evaluate the effects of a saffron extract on anxiety and depression following a wash-out period. Our results indicate a maintenance of the benefits on depression (BDI and HAMD), anxiety (STAI-S), emotion (positive and negative PANAS), and well-being (SWLS and Happiness Measure) for at least 2 weeks after cessation. The maintenance of the physiological effects of scutellaria extract after a wash-out period has not been investigated yet. Our findings demonstrated a maintenance of the effects of scutellaria extract on depression (HAMD), anxiety (STAI-S), and well-being (WHO-5 and Happiness Measure), for at least 2 weeks after cessation. A sustained effect was observed as well with the combination of both extracts in the SAFFR’UP group, which maintained the effects on depression (BDI, HAMD), anxiety (STAI-S), emotion (positive PANAS), and well-being (WHO-5, SWLS, and Happiness Measure). Further studies should determine whether the sustained effects of those two extracts might be further prolonged than the 2 weeks investigated here.

## 5. Conclusions

Overall, our results suggest that saffron and scutellaria extracts alone, and in combination, may offer promising benefits in the treatment of anxiety and depression. However, limitations of the present study must be considered. The participants had mild-to-moderate depression, and the effects of SAFFR’ACTIV^®^ and SCUTELL’UP^®^, and their combination, in individuals with severe depressive disorders remain unknown. The antidepressant effects were evaluated over a six-week supplementation period, highlighting the need for further research to assess the long-term safety and efficacy of these products, as well as the safety of higher doses beyond the commonly recommended ranges. Finally, the reliance on self-reported instruments may have resulted in measurement inaccuracies due to the subjective interpretation of items. Utilizing objective measures to assess stress hormone levels or neurotransmitter levels could help minimize potential errors.

In conclusion, this study offers further evidence of the beneficial effects of saffron extract on depression and anxiety in individuals with mild-to-moderate depression. Additionally, it highlights, for the first time in humans, the positive effects of scutellaria extract, as well as the combined effects of saffron and scutellaria extracts on both depression and anxiety.

## Figures and Tables

**Table 1:** Comparisons of the baseline characteristics between the SAFFR’ACTIV, SCUTTEL’UP, SAFFR’UP, and Placebo groups.

Variables	SAFFR’ACTIV Group (*n* = 43)	SCUTTEL’UP Group (*n* = 42)	SAFFR’UP Group (*n* = 48)	Placebo Group (*n* = 47)	
	Mean (SD)	Mean (SD)	Mean (SD)	Mean (SD)	*p*-Values
Age	34.0 (15.2)	38.3 (14.2)	33.3 (14.7)	35.6 (15.9)	0.410
Gender (M)	30.2%	26.2%	27.1%	29.8%	0.968
BMI	71.9 (14.8)	72.4 (14.5)	72.9 (15.7)	72.1 (14.2)	0.977
HR_rest_ (bpm)	72.2 (13.6)	72.5 (13.3)	77.5 (12.8)	73.7 (12.4)	0.178
BDI	20.9 (4.9)	23.1 (5.0)	21.6 (5.2)	21.6 (4.9)	0.236
HAMD	16.5 (3.3)	17.3 (3.0)	16.8 (3.1)	16.2 (3.3)	0.436

BDI, Beck Depression Inventory; BMI, body mass index; HAMD, Hamilton depression rating scale; HR_rest_, heart rate at rest; SD, standard deviation.

**Table 2:** Comparison of the questionnaire scores between the SAFFR’ACTIV, SCUTTEL’UP, SAFFR’UP, and Placebo groups at baseline (V1), after 21 days of intervention (V2), and after 42 days of intervention (V3).

**Variables** **Mean (SD)**	**SAFFR’ACTIV Group** **(*n* = 41)**			**SCUTTEL’UP Group** **(*n* = 42)**		
	**V1**	**V2**	**V3**	**V1**	**V2**	**V3**
BDI	21.2 (4.8)	14.9 (8.2) **	12.4 (8.6) **^,#^	23.1 (5.0)	17.0 (8.7) **	15.0 (8.1) **
HAMD	16.6 (3.4)	10.9 (4.5) **	8.5 (4.2) **^,#^	17.3 (3.0)	12.6 (4.4) **	10.7 (4.8) **^,#^
STAI-S	50.7 (8.8)	45.5 (10.2) *	42.2 (9.9) **^,#^	54.4 (9.1)	52.6 (10.2)	48.8 (11.0) **^,#^
PANAS Positive	22.1 (7.7)	23.9 (8.7) *	25.2 (9.4) **	21.5 (8.0)	22.2 (8.8)	22.6 (8.4)
PANAS Negative	20.2 (8.8)	17.1 (7.2) *	15.5 (6.5) **^,#^	21.6 (8.1)	19.7 (7.2) *	18.6 (7.3) *
SWLS	19.3 (6.6)	20.3 (6.8)	22.3 (7.3) **^,#^	16.3 (6.4)	17.0 (6.1)	17.7 (6.9) *
Happiness Measure	4.4 (1.9)	5.6 (1.9) *	6.5 (1.9) **^,#^	3.3 (1.8)	4.5 (2.1) *	5.1 (2.2) **
WHO-5	8.9 (3.5)	12.4 (4.4) **	13.3 (5.3) **	6.9 (4.3)	9.0 (4.9) **	10.9 (5.9) **^,#^
**Variables** **Mean (SD)**	**SAFFR’UP group** **(*n* = 48)**			**Placebo group** **(*n* = 46)**		
	**V1**	**V2**	**V3**	**V1**	**V2**	**V3**
BDI	21.6 (5.2)	15.3 (8.0) **	11.8 (7.2) **^,#^	21.5 (4.9)	14.3 (7.8) **	13.6 (8.9) **
HAMD	16.8 (3.1)	10.4 (4.7) **	8.8 (4.7) **	16.2 (3.3)	9.1 (4.1) **	9.4 (4.6) **
STAI-S	52.0 (9.3)	47.5 (12.0) *	43.1 (10.6) **^,#^	52.1 (10.3)	47.7 (10.0) *	46.2 (11.3) **
PANAS Positive	17.7 (8.6)	19.6 (9.4) *	21.1 (10.3) **^,#^	21.9 (7.8)	23.3 (8.1) *	23.7 (7.8) *
PANAS Negative	17.0 (8.9)	15.0 (8.7) *	13.4 (6.6) **	21.7 (7.8)	17.5 (7.6) *	17.4 (8.7) *
SWLS	17.5 (6.2)	19.8 (7.5) *	21.4 (7.4) **^,#^	17.7 (5.8)	19.5 (5.6) *	20.4 (6.2) *
Happiness Measure	4.1 (1.8)	5.6 (2.0) *	6.3 (1.9) **	4.0 (1.7)	5.7 (1.9) *	6.0 (2.1) *
WHO-5	8.1 (3.6)	11.2 (4.9) **	12.8 (4.8) **	8.8 (4.0)	12.9 (4.9) **	12.2 (4.9) **

* *p* < 0.05, ** *p* < 0.001 different from baseline within a group (paired *t*-tests). ^#^ *p* < 0.05 different between V3 and V2 within a group (paired *t*-tests).

## Data Availability

Data are available upon request by sending an e-mail to cicn@uclouvain.be.

## Supplementary Materials

The following supporting information can be downloaded at https://www.mdpi.com/article/10.3390/nu17050809/s1, Table S1. Score differences between D56 and V1 and between D56 and V3 in the SAFFR’ACTIV, SCUTTEL’UP, SAFFR’UP, and Placebo groups. Table S2. Comparisons of HAMD sleeping scores between the SAFFR’ACTIV, SCUTTEL’UP, SAFFR’UP, and Placebo groups at baseline (V1), after 21 days of intervention (V2), and after 42 days of intervention (V3). Table S3. Comparisons of V3–V2 BDI scores between the SAFFR’ACTIV, SCUTTEL’UP, SAFFR’UP, and Placebo groups as a function of gender (males vs. females). Figure S1. Molecule of safranal. Figure S2. Molecule of crocins. R and R1corresponding to glucosyl and hydroxyl function. Glucosyl and hydroxyl function corresponds to (Gen) gentiobiose; (Glc) glucose; (Nea) neaopolitanoside; and (Trig) triglucoside. Figure S3. Molecule of baicalin.
