# Supplementary material for: The Effects of Combined Scutellaria and Saffron Supplementation on Mood Regulation in Participants with Mild-to-Moderate Depressive Symptoms: A Randomized, Double-Blind, Placebo-Controlled Study
Source: Nutrients. 2025 Feb 26;17(5):809. doi: 10.3390/nu17050809 (PMC11901551; doi:10.3390/nu17050809)
Supplement: Supplementary file 1 [file nutrients-17-00809-s001.zip › nutrients-3462758-supplementary.pdf]

Supplementary tables and figures

1. Day 56 difference measurements

To assess the evolution of symptoms after stopping the intervention, participants completed the different questionnaires at D56 after inclusion. The descriptive statistics for the D56-V1 and D56-V3 score difference of the questionnaires are presented in the Table S1 hereafter.

Table S1. *Score differences between D56 and V1 and between D56 and V3 in the SAFFR'ACTIV, SCUTTEL'UP, SAFFR'UP and Placebo groups.*

**Variables** Mean (SD)	**SAFFR'ACTIV group** (n=39)		**SCUTTEL'UP group** (n=36)	
	**D56 – V1**	**D56 – V3**	**D56 – V1**	**D56 – V3**
BDI	-7.5 (8.9)**	1.0 (3.4)	-8.0 (7.6)**	-0.4 (5.3)
HAMD	-9.1 (5.2)**	-1.0 (4.0)	-7.9 (4.1)**	-1.4 (3.8)
STAI-S	-7.4 (12.9)*	1.2 (7.8)	-3.3 (9.0)*	1.9 (7.3)
PANAS Positive	2.2 (6.0)*	-0.5 (5.1)	0.6 (4.5)	-0.3 (3.4)
PANAS Negative	-3.9 (5.7)**	0.7 (3.7)	-1.4 (4.8)	0.7 (5.0)
SWLS	3.5 (5.2)**	0.5 (3.9)	1.7 (4.7)*	0.5 (4.6)
Happiness Measure	1.7 (2.5)**	-0.3 (1.5)	1.3 (1.4)**	-0.3 (1.9)
WHO-5	4.3 (5.7)**	0.0 (4.1)	3.2 (3.9)**	-0.7 (4.9)
**Variables** Mean (SD)	**SAFFR'UP group** (n=45)		**Placebo group** (n=38)	
	**D56 – V1**	**D56 – V3**	**D56 – V1**	**D56 – V3**
BDI	-8.6 (7.7)**	1.3 (6.9)	-7.3 (7.6)**	0.6 (4.4)
HAMD	-8.9 (5.4)**	-0.8 (4.1)	-7.7 (5.4)**	-1.1 (4.0)
STAI-S	-7.0 (11.4)**	1.9 (8.7)	-4.3 (8.0)*	0.6 (9.0)
PANAS Positive	2.8 (5.6)*	-0.7 (3.6)	-0.5 (4.3)	-1.7 (4.1)$^{\#}$
PANAS Negative	-1.8 (6.6)	1.7 (4.8)	-3.1 (5.5)*	0.9 (4.5)
SWLS	3.8 (4.1)**	-0.3 (2.9)	3.3 (4.6)**	0.6 (3.2)
Happiness Measure	1.9 (2.3)**	-0.3 (1.6)	1.5 (1.9)**	-0.3 (1.8)
WHO-5	5.0 (5.8)**	0.3 (3.9)	3.0 (4.5)**	0.1 (3.6)

* $p < 0.05$, ** $p < 0.001$ between D56 and V1 within a group (paired *t*-tests). $^{\#}$ $p < 0.05$ between D56 and V3 within a group (paired *t*-tests).

2. HAMD sleep items

Three specific items from the HAMD questionnaire that assess sleep dimensions—namely, difficulties in falling asleep (initial insomnia), interruptions during the night (middle insomnia), and early morning awakenings (terminal insomnia)—were identified. Additional analyses were performed by calculating the mean score of these three items to better evaluate the effects of the interventions on sleep disturbances, according to the intervention group. The descriptive statistics for the mean HAMD sleeping score are presented in the Table S2 hereafter.

Table S2. *Comparisons of HAMD sleeping scores between the SAFFR'ACTIV, SCUTTEL'UP, SAFFR'UP and Placebo groups at baseline (V1), after 21 days of intervention (V2), and after 42 days of intervention (V3).*

**Variables** Mean (SD)	**SAFFR'ACTIV group** (n=41)			**SCUTTEL'UP group** (n=42)		
	**V1**	**V2**	**V3**	**V1**	**V2**	**V3**
HAMD Sleeping items	0.98 (0.37)	0.72 (0.46)	0.57 (0.42)$^{\#}$	1.11 (0.37)	0.90 (0.56)	0.83 (0.50)
**Variables** Mean (SD)	**SAFFR'UP group** (n=48)			**Placebo group** (n=46)		
	**V1**	**V2**	**V3**	**V1**	**V2**	**V3**
HAMD Sleeping items	1.08 (0.44)	0.67 (0.53)	0.62 (0.47)	1.01 (0.49)	0.58 (0.43)	0.60 (0.50)

$^{\#}$ $p < 0.05$ different from V2 within a group (paired *t*-tests).

3. Gender effect for the V3-V2 BDI score difference

Separate exploratory analyses were conducted for men and women to explore potential gender-specific effects, according to the intervention group. These analyses suggested a specific effect for women on the primary outcome variable, namely the BDI score. The descriptive statistics for the V3-V2 BDI score difference as a function of gender are presented in the Table S3 hereafter.

Table S3. *Comparisons of V3-V2 BDI scores between the SAFFR'ACTIV, SCUTTEL'UP, SAFFR'UP and Placebo groups as a function of gender (males vs females).*

**Variables** Mean (SD)	**SAFFR'ACTIV group** (n=41)	**SCUTTEL'UP group** (n=42)
	**V3-V2**	**V3-V2**
BDI score		
Males	-3.6 (5.5)	-1.5 (10.3)

Females	-1.9 (5.8)	-2.2 (6.5)

**Variables**<br>Mean (SD)	**SAFFR’UP group**<br>(n=48)	**Placebo group**<br>(n=46)
	**V3-V2**	**V3-V2**
BDI score		
Males	-3.1 (5.4)	-1.6 (6.2)
Females	-3.6 (7.1)*	-0.3 (6.8)

* $p < 0.05$ between V3 and V2 within a group (paired *t*-tests).

4. Figure of the three active compounds

SAFFR’ACTIV® is standardized in two active compounds, crocins and safranal.

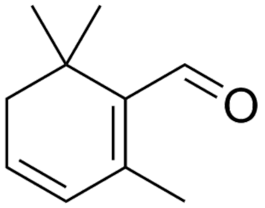

Figure S1. *Molecule of safranal*.

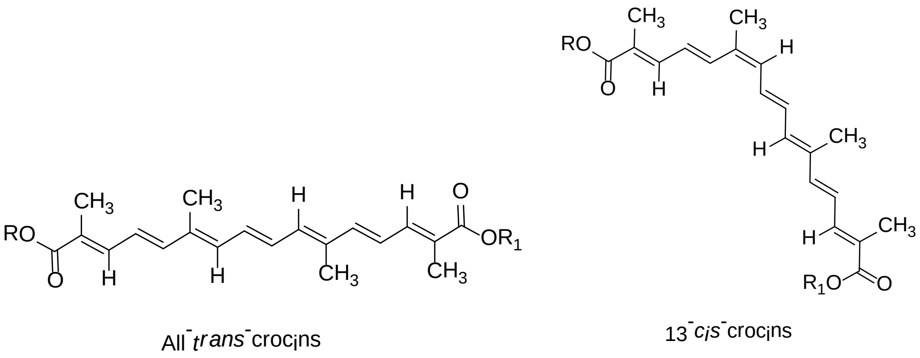

Figure S2. *Molecule of crocins. R and R1corresponding to glucosyl and hydroxyl function. Glucosyl and hydroxyl function corresponds to (Gen) gentiobiose; (Glc) glucose; (Nea) neaopolitanoside; and (Trig) triglucoside.*

SCUTELL’UP® is standardized in baicalin.

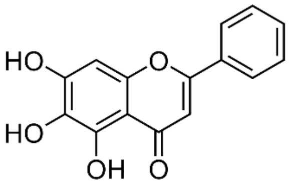

Figure S3. *Molecule of baicalin.*
